# Editorial: Addressing the sexually transmitted infections epidemic in the United States: a sociomedical perspective

**DOI:** 10.3389/fpubh.2023.1320080

**Published:** 2023-11-06

**Authors:** Christopher Williams, Keith L. Gray, Laura A. Skrip, Darren L. Whitfield

**Affiliations:** ^1^Department of Natural and Social Sciences, State University of New York at Purchase College, Purchase, NY, United States; ^2^Ministry of Health, Monrovia, Liberia; ^3^School of Public Health, University of Liberia, Monrovia, Liberia; ^4^Quantitative-Data for Decision Making Lab, University of Liberia, Monrovia, Liberia; ^5^School of Social Work, University of Maryland Baltimore, Baltimore, MD, United States

**Keywords:** intimate partner violence, chemsex, STI screening and at-home testing, sexual health indicators, HIV-1 recombinants

Sexually transmitted infections (STIs) cause significant morbidity and mortality in the United States (US) and across the globe. Within the last decade, there have been substantial advances in the number and diversity of biomedical and public health interventions critical for controlling STIs. Despite these gains, incident STIs are on an upward trajectory (1). In 2022 there were ~1.3 million new STIs equating to 3,500 new infections globally per day (2). STIs have a direct impact on acute and chronic health outcomes and are implicated in neurological and cardiovascular diseases, cancer, infertility, and pregnancy complications (3). According to a seminal report by the US National Academies of Sciences, Engineering, and Medicine, the STI epidemic exacts a deleterious medical and economic toll on individuals and society as a whole (4). And although STIs are pervasive, poor and disadvantaged populations bear a disproportionate share of disease burden (5).

The STI epidemic ranks high among the most pressing public health concerns and has captured attention across disparate research disciplines. This widespread interest is reflected in the fact that 13 peer-reviewed articles were published, which to date had 20k views and downloads. This Research Topic contains methodologically rigorous research approaches including qualitative and quantitative data analytic strategies, proof-of-concept and efficacy study designs, and systematic, meta-analytic, and bibliometric reviews. Each article advances the science in its respective field and collectively they inform hypothesis generation, encourage replication studies, and support clinical practice and public health policy decision-making. The impetus for this Research Topic stems from a recognition that the STI epidemic is driven by a complex interaction of biological, behavioral, and structural determinants of disease. As such, manuscripts involving innovative screening and diagnostic tools, high-priority populations, and public health policies and practices were prioritized.

Chlamydia, herpes, gonorrhea, and syphilis infections are key risk factors for other more consequential pathogens including HIV. Evidence from epidemiological studies has shown that ~1 of 10 HIV infections is directly attributable to incidence of the most common STIs (6). And although there has been a number of innovations in HIV/AIDS care, it remains a serious public health threat made worse by increasing global genetic diversification. Nchinda et al. explores this diversity in their systematic review of HIV-1 recombinants among highly-vulnerable populations. They report that injection drug use was associated with the greatest odds of recombinants and circulating recombinant forms while commercial sex work was associated with increased odds of recombinants and unique recombinant forms. These findings underscore the critical need for rigorous HIV-1 molecular surveillance as pathogens and new epidemiological patterns emerge. In a second review article, Nguyen et al. employed bibliometric techniques (e.g., topic modeling) to examine knowledge gaps in HIV/AIDS research. Their work identified growing interest in research that examines the complex dynamics of intimate partner violence and HIV/AIDS as well as a dearth of research on pregnant women and at-risk adolescents.

Men who have sex with men (MSM) are another high-priority population warranting renewed multidisciplinary research consideration. “Chemsex,” a type of high-risk prolonged sexual activity practiced among a subpopulation of MSM, involves intentional drug use before or during sessions with multiple known and unknown partners. Unfortunately, few harm-reduction interventions to address chemsex have been rigorously evaluated (7). In this Research Topic, Platteau et al. tested a mobile phone application for its efficacy in reducing substance use (e.g., use of GHB, methamphetamine). The app showed promise for relaying messages to and from researchers and for promoting individualized harm reduction strategies. Vallée's review paper also considered chemsex with more commonly available substances. Their analysis indicated that cannabis and alcohol use were linked to an increased number of sexual partners and decreased immune response to monkey pox infection—demonstrating the importance of rapid knowledge transfer at the earliest stage of STI outbreaks.

Widespread availability of effective screening and diagnostic tools are essential for advancing toward an end to global STI epidemics; however, adoption of innovative tools and good clinical practices has not kept pace with the dispersion and prevalence of the most common infections (8). Telehealth medicine holds significant promise toward this goal. The next three articles make important contributions in this area. For instance, Zoschke et al. assessed screening practices of healthcare providers who serve MSM—a population at increased risk for human papillomavirus-associated oropharyngeal cancer. Overall, providers reported low self-confidence and hesitancy in screening for oropharyngeal cancer; however, those who practiced in LGBTQ+ specialty healthcare reported less hesitancy and expressed more confidence and skill in conducting screenings. In a similar population, Ross et al. examined the efficacy of “selfies”—pictures taken at home by patients—of the oral cavity. Patients who submitted selfies were younger, more knowledgeable about HPV, and more likely to have had an HPV vaccine than those who did not. Furthermore, provider inter-rater agreement about image quality to screen for oral malignances was determined to be acceptable. In another study examining telehealth practices, Van Gerwen et al. assessed attitudes toward at-home collection of gonorrhea and chlamydia specimens among transgender women. Preliminary evidence suggests that participants were enthusiastic about at-home extragenital STI testing—a finding that has important implications for avoiding provider-driven stigma and mitigating gender dysphoria. Combined, these studies point to the impact of rapid, affordable, easy-to-use testing innovations that are rigorously evaluated in high-priority populations.

For cisgender reproductive-aged women, even the most common STIs can have a consequential impact. Li and Chen's work is important in this regard as it extends basic science research demonstrating the association between infertility and Pgp3AbMBA antibody levels—a scarcely-used assay for chlamydia. Their findings indicated that each standard increment in Pgp3AbMBA predicted a substantial increase in infertility and this association persisted when limiting the analysis to women who had previously given birth. Stapleton et al. also investigated chlamydia infection, but with a sample of carceral-involved men who have sex with women. Multivariate analyses revealed that having a history of incarceration was positively associated with a positive chlamydia test even after adjusting for potential confounders (e.g., insurance status). This article contributes to a growing body of research demonstrating the shared social and structural determinants of STI acquisition and incarceration.

The development of innovative behavioral interventions to reduce STIs is critical for reducing STI acquisition (4). Using a mixed-method approach including survey data and qualitative assessments, Filippone et al. found that motivational interviewing and monetary incentives were both feasible and efficacious in suppressing HIV viral load in a sample of patients from low-income backgrounds—demonstrating the value of behavioral interventions that consider financial hardship as a proximal barrier to viral load suppression.

Vaccination, a type of prevention as intervention, is an important tool in the arsenal for controlling STIs. The availability of safe and effective vaccines has increased; however, few countries have reached adequate vaccination rates (9). Dykens's et al. article is important in this regard as they critically review policies to expand gender-neutral HPV vaccination programs so that boys are vaccinated at rates comparable to girls. Their work suggests that a more comprehensive approach to HPV vaccine programing is essential for reducing morbidity and would also have the added benefit of reducing misinformation and vaccine-related stigma. In addressing HPV vaccine hesitancy, Moya et al. conducted qualitative assessments among community health workers and healthcare providers who serve Latino people. They identified financial concerns, misinformation, and entrenched cultural norms and attitudes as salient barriers to broad-scale HPV vaccination.

To effectively address STI epidemics, it is essential to develop a comprehensive sexual health approach informed by a multidisciplinary body of stakeholders. Ford et al. examined various national surveys and surveillance systems for the development of a national score card on the state of sexual health in the US. Their work identified four broad domains including knowledge, behaviors and relationships, service access and utilization, and adverse health outcomes as essential in measuring and monitoring core sexual health indicators. This work holds promise for a national strategy to promote holistic sexual health.

Collectively, these articles highlight the complex interaction between social networks, infectious pathogens, epidemiological patterns, and a myriad of structural forces linked to STI morbidity and mortality. This Research Topic is published at a time of increasing STI incidence and eroding public health infrastructure and these factors are made worse by emergent global antimicrobial resistance. In order to achieve the World Health Organization Sustainable Development Goal of eliminating STI epidemics as major health threats, a renewed commitment to cutting-edge research and rapid transfer of innovation to practice is warranted (10). The articles presented here are an important step toward this public health imperative.

## Author contributions

CW: Conceptualization, Writing—original draft. KG: Writing—review & editing. LS: Writing—review & editing. DW: Writing—review & editing.
